# The origin and evolution of HKT proteins with TrkH domain from aquatic plants to flowering plants

**DOI:** 10.1093/hr/uhaf245

**Published:** 2025-09-15

**Authors:** Ping Li, Runzhe Hu, Yazhou Zhao, Wentong Liu, Qin Zhang, Tangchun Zheng, Yun Wu, Yinran Huang

**Affiliations:** College of Landscape and Tourism, College of Forestry, Hebei Key Laboratory of Floral Biological Breeding, Hebei Agricultural University, Baoding 071000, China; College of Landscape and Tourism, College of Forestry, Hebei Key Laboratory of Floral Biological Breeding, Hebei Agricultural University, Baoding 071000, China; College of Landscape and Tourism, College of Forestry, Hebei Key Laboratory of Floral Biological Breeding, Hebei Agricultural University, Baoding 071000, China; College of Landscape and Tourism, College of Forestry, Hebei Key Laboratory of Floral Biological Breeding, Hebei Agricultural University, Baoding 071000, China; College of Landscape and Tourism, College of Forestry, Hebei Key Laboratory of Floral Biological Breeding, Hebei Agricultural University, Baoding 071000, China; National Engineering Research Center for Floriculture, School of Landscape Architecture, Beijing Forestry University, Beijing 100083, China; Discipline of Ornamental Horticulture, College of Horticulture Science, Zhejiang A&F University, Hangzhou 311300, China; College of Landscape and Tourism, College of Forestry, Hebei Key Laboratory of Floral Biological Breeding, Hebei Agricultural University, Baoding 071000, China

## Abstract

HKT proteins are the conserved voltage-gated ion channel proteins that act on the transmembrane transport of positive ions, but the evolutionary history of the HKT gene family is not clear. To create comparative HKT resources, we collected 134 HKT sequences in different phylogenetic lineages ranging from algae to angiosperms, encompassing fifty distinct taxonomic species. The evolutionary history of the HKT gene family was revisited through phylogenetic reconstruction. Phylogenetic reconstruction and comparative genomic analysis suggested that the HKT gene family originated from land plants and had a large number of tandem duplications. The evolution of *HKT* genes was mostly linear in non-seed plants. The genome duplication event was a potential factor in the change of the *HKT g*ene copies in seed plants. In eudicots, the contraction event of *HKT* genes occurred every time the plant underwent a whole-genome duplication event after the ancient triplication. Amino acid sequence variations in the HKT transporters of TrkH domain influence their tertiary protein structure. Meanwhile, six *HKT* genes, represented by the *Vitis vinifera*, exhibit tissue-specific expression patterns and respond differentially to salt and drought stress. Frequent gene and genome duplications contributed significantly to the expansion/contraction of the HKT gene family. Our findings regarding the origin and evolution of *HKT* offer unique backgrounds and new insight into the functional evolution of this gene family.

## Introduction

HKTs are the conserved voltage-gated ion channel (VIC) proteins that act on the transmembrane transport of positive ions, including sodium (Na^+^)/potassium (K^+^) homeostasis, Na^+^ and K^+^ uptake [1]. HKT family consists of various cation transport proteins and V-type Na^+^ ATP synthase subunit J or translocating ATPase J, and HKTs are involved in active Na^+^ and K^+^ uptake utilizing ATP [2, 3]. HKT proteins have the conserved TrkH domain and determine the specificity and kinetics of cation transport by the Trk system in this organism [3, 4].

In previous studies, HKT sequences are identified in bryophytes, lycophytes, and euphyllophytes [1]. Although HKT is widely studied in various plants, the number of species involved is very small due to the lack of plant genomic data. In older species, HKTs are only identified in *Physcomitrella patens* belonging to bryophytes and *Selaginella moellendorffii* belonging to lycophytes [1, 5]. The HKT (MA_54668g0010) sequence is found in gymnosperms represented by *Picea abies* [1]. Among monocotyledons, the study of *HKT* genes in rice are the most detailed and seven HKT sequences are identified in *Oryza sativa* subsp. Japonica [1, 6–8]. Only one HKT sequence is identified in eudicots represented by *Arabidopsis thaliana* [9, 10]. Even among euphyllophytes, systematic analyses of HKT family members remain scarce, with research efforts predominantly concentrated on characterizing individual *HKT* gene functions.

HKT proteins share a common structure of four transmembrane domain-pore domain-transmembrane domain units (MPM1-MPM4) [1, 11–13]. The HKT transporter family is phylogenetically divided into two distinct subgroups (types I and II) based on structural characteristics [5, 11, 13]. I type and II type HKTs are differentiated by the presence of either a serine residue or a glycine residue in the first pore loop [10, 11, 13]. The sequences of I type HKT show more diversity, while, the pore domain’s helical region sequences of II type HKTs show a higher grade of conservation [1]. I type and II type HKTs translate into class-specific differences in ion conduction. AtHKT1, belonging to type I HKT, lacks a conserved Gly at position 68 [10]. AtHKT1 does not transport K^+^ but regulates K^+^ nutrient status via its ability to facilitate Na^+^ homeostasis [10, 14]. *O. sativa* includes two type of HKT transporters, a Na^+^ transporter (I type) and a Na^+^- and K^+^-coupled transporter (II type) [8]. This classification of HKT is limited to angiosperms and more concentrated in monocotyledons. Only I type HKTs are identified in eudicots so far [1].

The domain of HKT protein is up to 502 amino acids (aa) from the Pfam database [15]. The sequence of the HKT type can be identified in genome-wide by several conserved consensus motifs [13]. During the long run road of evolution, different species have a specific number of HKT sequences and each HKT has sequence differences [16, 17]. The most recent common ancestor of all embryophytes comprised a single protein of the HKT type [13]. *P. patens* (bryophytes) has a single extant representative (HKT, Pp3c1 15 810 V3.1.p), whereas several duplication events occur in different lineages in tracheophytes [13]. Previous studies have reported that II type HKT is identified in all species, whereas I type HKT is found only in small grain cereals. An ancestral gene duplication postdivergence from the coarse grain cereal lineage may be the reason for the formation of I type HKT [13]. The subfamily division of HKT emerges in land plants only after the separation of lycopodiophytes [1, 13]. Meanwhile, many studies have found exceptions to this classification [5, 16, 18–20]. Consequently, this classification system demonstrates limited applicability to the comprehensive spectrum of plant *HKT* genes.

The evolutionary journey of plants, from aquatic origins to terrestrial colonization and ultimately to the emergence of flowering plants, spans approximately several billion years [21, 22]. The relationship between plants and their environment manifests through multidimensional interactions, while plants continuously reshape their surroundings through evolutionary adaptations [23]. Environmental selective pressures drive changes in gene frequencies, and innovations in gene function further expand the ecological niches of plants [24]. Alterations in plant genes constitute a pivotal element in deciphering the evolutionary trajectory of botanical lineages. In recent years, the release and open access to a substantial number of plant genomes spanning diverse phylogenetic lineages, coupled with the establishment of well-curated databases, have significantly enhanced gene mining efficiency and utilization [25–29]. Concurrently, the development of various functional annotation databases has substantially advanced our understanding of nearly all aspects of plant biological functions [30–33]. These decoded genomes bridge the huge evolutionary gap between different phylogenetic lineages and provide a large amount of data support for solving some of the major questions in species and gene evolution. In this study, we investigated the evolutionary history of *HKT* genes in all major plant lineages using gene structural information, phylogenetic analyses and bioinformatics tools. Our study identified *HKT* genes in plants and revealed the origin and evolutionary pattern of *HKT* genes.

## Results

### Origin and evolution of the plant *HKT* genes

**Figure 1:**
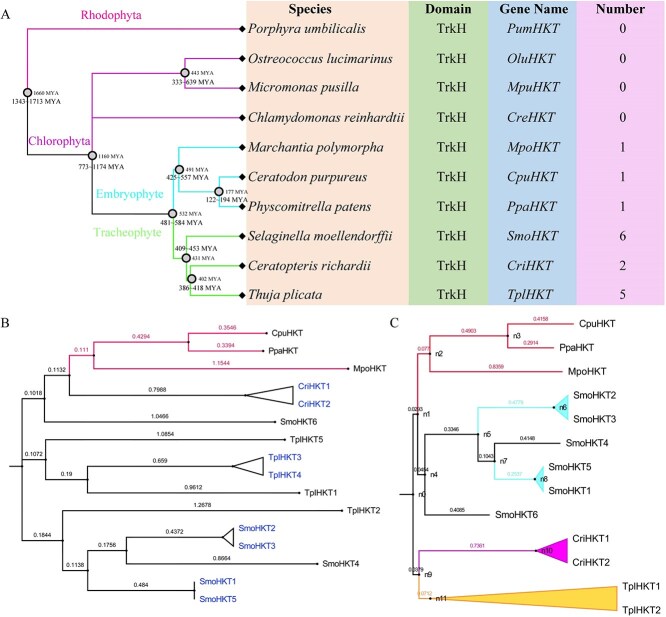
Identification and phylogeny of HKT gene family in the ancient plants. A. Phylogeny and number of HKT gene family in ten species. The estimation of plant divergence times was calibrated with reference to the fossil records of relevant species. B. Phylogenetic tree of HKT protein sequence from three embryophytes and three tracheophytes. C. Genome-wide identification of orthogroups and hierarchical orthogroups of HKT gene families in three embryophytes and three tracheophytes.

**Figure 2:**
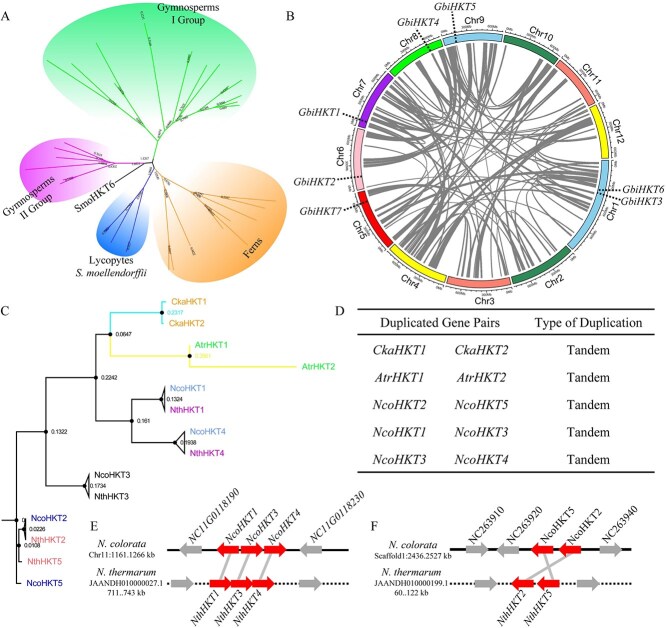
Phylogeny and duplication events of HKT gene family in gymnosperms and basal angiosperms. A. Phylogeny of HKT gene family in six gymnosperms, three ferns, and one lycophytes. B. Synteny genes of paralogous sequences related to the WGD event in *G. biloba*. C. Phylogeny of HKT gene family in four basal angiosperms. Words marked with the same color represented potentially duplicated genes, except black. D. Tandem duplication of gene pairs. E and F. Chromosomal distribution and potentially collinearity duplication of *HKT* genes in *N. colorata* and *N. thermarum*.

In ten plants, a total of sixteen *HKT* genes were identified, one in *P. patens* (bryophyte), one in *Ceratodon purpureus* (bryophyte), one in *Marchantia polymorpha* (liverwort), two in *Ceratopteris richardii* (fern), five in *Thuja plicata* (gymnosperm), and six in *S. moellendorffii* (lycophyte) (Fig. 1 and Table S3). Interestingly, *HKT* genes were not detected in the ancient plants rhodophyte (*Porphyra umbilicalis*), and chlorophytes (*Ostreococcus lucimarinus*, *Micromonas pusilla,* and *Chlamydomonas reinhardtii*). The Rhodophyta and Chlorophyta were species-rich ancient groups of photoautotrophic organisms with fossil ages of approximately 1660 million years ago (MYA) and 1160 MYA, respectively. The speciation times approximately were 481 – 584 MYA for the divergence between embryophytes and tracheophytes. We found only one *HKT* gene in every embryophyte plant, whereas two or more *HKT* genes in every tracheophyte plant. The *HKT* genes appeared during the evolution of plants from aquatic to terrestrial, which also indicated that *HKT* genes played a potentially important role in plant adaptation to the terrestrial environment. Comparative phylogenetic analysis revealed strong evolutionary conservation of HKT gene sequences across embryophyte and tracheophyte lineages (Figs 1B and 2C). In tracheophytes, four pairs of tandemly duplicated *HKT* genes were identified in the genome, one in *C. richardii* (*CriHKT1* and *CriHKT2*), one in *T. plicata* (*TplHKT3* and *TplHKT4*) and two in *S. moellendorffii* (*SmoHKT2* and *SmoHKT3*, *SmoHKT1*, and *SmoHKT5*) (Fig. 1B and Table S4). Meanwhile, one collinearity duplicated *HKT* genes (*SmoHKT2* and *SmoHKT5*) were identified in *S. moellendorffii* genome (Table S4). In *S. moellendorffii*, the consistency of the two tandemly duplicated *HKT* gene pairs was 92.32% and 91.77%, respectively. We inferred that the *SmoHKT* genes first underwent collinearity duplication event and then tandem duplication event, contributing to the expansion of gene families. OrthoFinder assigned 277, 215 genes (87.6% of total) to 33, 633 orthogroups. Fifty percent of all genes were in orthogroups with 12 or more genes (G50 was 12) and were contained in the largest 7015 orthogroups (O50 was 7015). In *T. plicata*, *TplHKT3* and *TplHKT4* were separately assigned to an orthogroup, and *TplHKT5* was an unassigned gene according to Markov Cluster Algorithm (MCL). Phylogenetic hierarchical orthogroups (HOG) showed that *CpuHKT* and *PpaHKT* were HOG and they evolved from a common ancestor. *SmoHKT* experienced three duplication events (n4) and formed six *SmoHKT* paralogous genes (Fig. 1C).

### Evolution of the ferns, gymnosperms, and basal angiosperms *HKT* genes

The morphology of the fern spores was stable and their ornamentations had important value in the systematic evolution and taxonomy of pteridophytes. At present, the genomes of three ferns have been published, namely *C. richardii*, *Azolla filiculoides,* and *Salvinia cucullata*. Here, a total of ten *HKT* genes were identified from their genomes (Figs 2A, S1, and Table S3). All *HKT* genes of ferns were divided into one branch and had a closer evolutionary distance from the *HKT* genes of lycophytes than gymnosperms (Figs 2A and S2).

**Figure 3:**
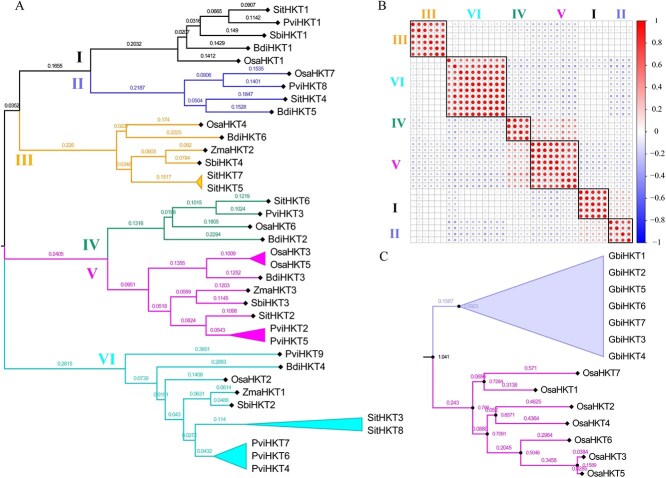
Identification and phylogeny of HKT gene family in the monocotyledons. A. Phylogeny and number of HKT gene family in six monocotyledon species. B. Correlation and cluster analysis of HKT sequence consistency. C. Phylogenetic tree of HKT protein sequence from *G. biloba* and *O. sativa*.

In gymnosperms, we identified a total of twenty-two HKT protein sequences, five in *T. plicata,* seven in *Ginkgo biloba*, five in *Gnetum montanum*, two in *Abies alba*, one in *Pinus abies*, one in *P. lambertiana*, and one in *P. tabuliformis*. The HKT sequences of gymnosperms were divided into two groups, of which HKT sequences of II Group were clustered with the lycophytes and ferns. One or two HKT sequences were identified in each Pinaceae species and clustered into one branch. The *G. biloba* was the only living representative of the order Ginkgoales, a group of gymnosperms dating back to 270 MYA in the Permian period. The whole genome of *G. biloba* was published in the public database, so we identified and analyzed the *HKT* genes with *G. biloba* as the representative of gymnosperms. Here, we identified seven *HKT* genes from ginkgo genome, which were randomly distributed on six chromosomes (Figs 2A, 3B, and Table S3). Phylogenetic analysis indicated that seven GbiHKT sequences were divided into three directions from a common ancestor. *GbiHKT5* occupied a direction as an independent member (Fig. 2A). As a direct member, the consistency of *GbiHKT1* and *GbiHKT2* had the highest homology, but the sequence identity was only 59.27%. *GbiHKT3*, *GbiHKT4*, *GbiHKT6,* and *GbiHKT7* were assigned to the same branch, and *GbiHKT3* and *GbiHKT6* genes were located in the 429.1 to 452.5 Mb range on chromosome. 1411 collinearity and 3126 tandem genes were identified from the *G. biloba* genome, but no *GbiHKT* genes were retrieved among them (Fig. 2B).

Basal angiosperms were the first flowering branch of the primitive angiosperms. *Amborella trichopoda*, *Cinnamomum kanehirae*, *Nymphaea colorata,* and *N. thermarum* were extant representatives of lineages that diverged the earliest from the lineage leading to the extant mesangiosperms. A total of fourteen *HKT* genes were identified in four basal angiosperms, two in *A. trichopoda*, two in *C. kanehirae*, five in *N. colorata*, and five in *N. thermarum* (Fig. 2C and Table S3). The *HKT* genes were tandemly duplicated genes in *A. trichopoda* and *C. kanehirae* (Figs 2C and 3D). Meanwhile, three pairs of tandemly duplicated *HKT* genes were identified in the *N. colorata* genome (Figs 2C and 3D). Because the *N. thermarum* genome could not be assembled to the chromosome level, duplicate genes were not identified. However, we found that *NthHKT* genes had high homology with *NcoHKT* genes and were anchored to two scaffolds (Figs 2E and 3F). Comparative genomic analysis suggests that *NthHKT* genes likely underwent duplication events analogous to those observed in *NcoHKT* genes. The seven *GbiHKT* genes formed an independent branch between *G. biloba* and four basal angiosperms (Fig. S2). Phylogenetic analyses revealed significant sequence conservation of *HKT* genes across both gymnosperm and basal angiosperm lineages.

### Identification and phylogeny of HKT gene family in the monocotyledons

**Figure 4:**
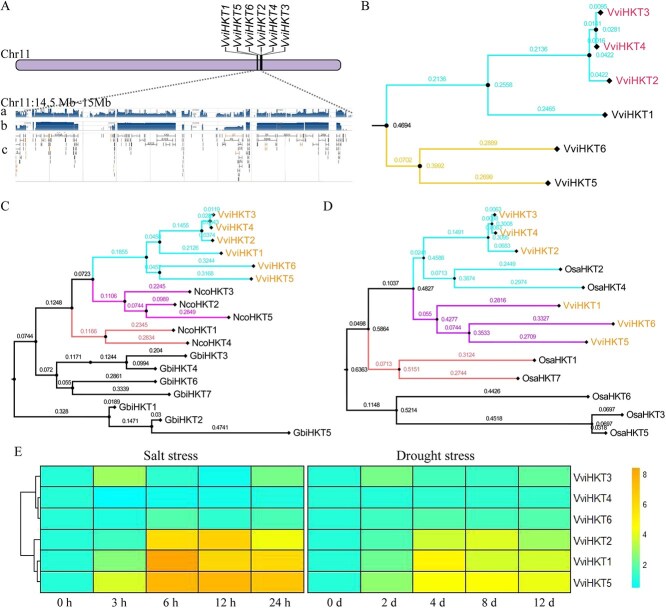
Identification and phylogeny of HKT gene family in the *V. vinifera*. A. Chromosome location and structural characteristics of *VviHKT* genes. a. RNA-seq exon coverage and aggregate (filtered). b. RNA-seq intron-spanning reads and aggregate (filtered). c. RNA-seq intron features and aggregate (filtered). B. Phylogeny of HKT gene family in *V. vinifera*. C. Phylogenetic tree of HKT protein sequence from *G. biloba, N. colorata*, and *V. vinifera*. D. Phylogenetic tree of HKT protein sequence from *O. sativa* and *V. vinifera*. E. Expression patterns of *VviHKT* genes in response to salt and drought stress.

We identified thirty-seven *HKT* genes in six monocotyledons, seven in *O. sativa*, three in *Zea mays*, four in *Sorghum bicolor*, eight in *Setaria italica*, nine in *Panicum virgatum,* and six in *Brachypodium distachyon* (Fig. 3 and Table S3). Thirty-seven *HKT* genes were divided into six groups (i.e. I, II, III, IV, V, and VI). The clustering results of gene sequence consistency showed that *HKT* genes were divided into six groups, which were consistent with the phylogenetic grouping, and the consistency of gene sequences in each group was highly correlated (Figs 3A and 4B). Interestingly, we found that although the number of *HKT* genes varies greatly in monocotyledons, *HKT* genes were not obviously clustered according to species (Fig. 3A). The monocotyledons whole-genome duplication (WGD) events followed by diploidization likely drove selective losses of HKT genes, while the aquatic adaptation history of *O. sativa* appeared to have favored evolutionary retention of more HKT gene family members. Taking *O. sativa* as an example, *HKT* genes formed two independent branches between *O. sativa* and *G. biloba* (Fig. 3C). These results suggested that the expansion and contraction of HKT gene family occurred synchronously in monocotyledons. We identified seven pairs of tandemly duplicated *HKT* genes, one in *O. sativa*, one in *B. distachyon*, two in *P. virgatum,* and three in *S. italica* (Table S5). Here, *P. virgatum* was tetraploid (4×), and two collinearity duplicated gene pairs were identified from chromosomes labelled K and N (Chr4K vs. Chr4N and Chr7K vs. Chr7N), respectively (Fig. S3). Tandem duplication events were one of the reasons for the expansion of the HKT gene family in some monocotyledons.

### Identification and phylogeny of HKT gene family in the eudicots

To investigate the evolutionary characteristics of *HKT* genes in the eudicots genome, we selected twenty-two eudicots, including Brassicaceae (*A. thaliana*), Polygonaceae (*Fagopyrum esculentum*), Euphorbiaceae (*Speranskia yunnanensis*), Malvaceae (*Gossypium lobatum, G. schwendimanii,* and *G. klotzschianum*), Salicaceae (*Populus trichocarpa*), Vitaceae (*V. vinifera*), Rosaceae (*Prunus persica, P. armeniaca, P. mume, P. avium, Malus domestica, Pyrus communis, P. betulifoli, Rosa chinensis, Rubus occidentalis,* and *Fragaria vesca*) and Rutaceae (*Citrus sinensis, C. clementina, C. grandis,* and *C. ichangensis*) (Tables S6 and S3). Here, we identified a total of forty-one *HKT* genes and found that the number of *HKT* genes in each species was 1 or 2, except *F. vesca* and *V. vinifera*. Chromosome-scale comparative genomics identified significant intrageneric conservation of *HKT* gene numbers and chromosomal positions, with Malvaceae members *G. schwendimanii* and *G. klotzschianum* representing phylogenetic outliers showing disrupted syntenic conservation.

The *HKT* genes of *G. schwendimanii* and *G. klotzschianum* did not exist in the form of gene clusters, and *GscHKT2* and *GklHKT2* were separately divided into a branch in the phylogenetic tree compared with *HKT* genes of other eudicots (Fig. S4). Compared to *O. sativa, N. colorata,* and *G. biloba*, the *HKT* genes of all *Gossypium* were clustered into a branch (Figs S4 and S5). The results showed that *GscHKT2* and *GklHKT2* genes were formed with the evolution of *Gossypium*. In Rosaceae, *HKT* genes existed in clusters, and *HKT* genes were usually tandemly duplicated genes in species with more than two *HKT* genes. We found that four *FveHKT* genes were identified in *F. vesca*, of which only one tandemly duplicated gene pair (*FveHKT1* and *FveHKT2*) was present (Table S6). However, these genes were located in the range of 5.409 to 5.442 Mb on chromosome 4 (Table S7). The majority of the species in the *Malus* and *Pyrus* were diploid, with a haploid number of *n* = 17. Surprisingly, only one *HKT* gene was identified in *M. domestica, P. communis,* and *P. betulifoli*, respectively. The *V. vinifera* genome had not undergone recent genome duplication, and this ancestral arrangement was common in many dicotyledonous plant species. Six *VviHKT* genes were identified in the *V. vinifera* genome, and were located on chromosome 11 (14.5 – 15.0 Mb) (Table S6 and Fig. 4A). Six *VviHKT* genes were divided into two branches, and two pairs of tandemly duplicated genes (*VviHKT2* and *VviHKT4*; *VviHKT3,* and *VviHKT4*) were identified (Table S6 and Fig. 4B). Compared to *N. colorata* and *G. biloba*, the *VviHKT* genes were clustered into a branch (Fig. 4C). The *VviHKT1, VviHKT5,* and *VviHKT6* genes were separated by the *OsaHKT2* and *OsaHKT4* genes (Fig. 4D). We speculated that recent genome duplication event was responsible for the contraction of HKT gene family in other eudicots. Through the analysis of gene expression patterns, we found that the *VviHKT* genes were primarily expressed in root and stem tissues (Fig. S6). Overall, the *VviHKT* genes exhibited up-regulated expression in response to salt and drought stress, particularly *VviHKT1* and *VviHKT5* (Fig. 4E).

### Species distribution and structure characteristics of HKT proteins

**Figure 5:**
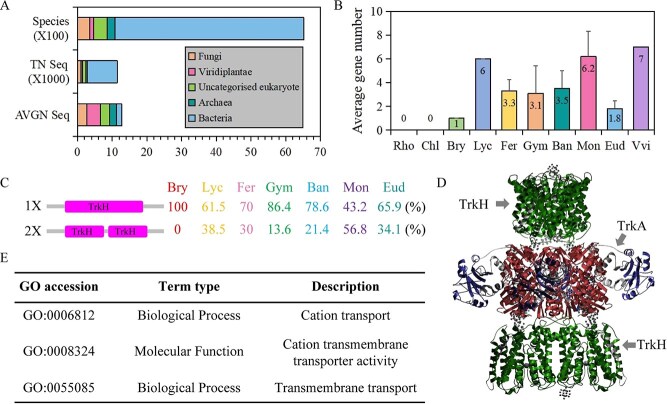
Species distribution and structure characteristics of HKT proteins. A. The distribution of Trk family across species based on the Pfam database. B. The average number of *HKT* genes in each plant category. C. The architecture analysis of 134 sequences from fifty species. D. Crystal structure of the TrkH/TrkA potassium transport complex (PDB entry 4J9U). E. Gene ontology and GO annotations about 134 *HKT* genes from fifty species. Rho represented rhodophytes; Chl represented chlorophytes; Bry represented embryophytes; Lyc represented lycophytes; Fer represented ferns; Gym represented gymnosperms; Ban represented basal angiosperms; Mon represented monocotyledons; Eud represented eudicots; Vvi represented *V. vinifera*.

To further investigate the species distribution of TrkH domain proteins, we further analyzed the distribution of Trk domain proteins in bacteria, archaea, and eukaryotes based on the Pfam database. Here, 12 573 sequences were identified in 5983 species, 935 in 345 fungi, 464 in 121 viridiplantae, 1023 in 386 uncategorized eukaryotes, 441 in 226 archaea, and 8640 in 5447 bacteria (Fig. 5A). The members of the Trk family were derived from Gram-negative and Gram-positive bacteria, yeast, and plants. We could find a representative list of sequences belonging to the proteins of the Trk family in the Transporter Classification Database. The members of Trk family were 1 or 2 in each bacterium and archaea species. However, there were an average of 3.1 Trk proteins per species in eukaryotes, with the largest average number per viridiplantae species (average 3.8 per viridiplantae species) (Fig. 5A).

Based on the fifty species selected in this study, we found that the average number of HKT sequences in monocotyledons was the largest. However, there were an average of 1.8 HKT sequences per species in eudicots with the removal of *V. vinifera* (Fig. 5B). Statistical analysis of HKT sequences showed that there were obvious differences among species in gymnosperms, basal angiosperms, and monocotyledons. Here, 134 sequences from fifty species had two types of domain architectures (TrkH x1 or TrkH x2) (Fig. 5C). The finding that HKT proteins in tracheophytes exclusively possess the TrkH_x1 type domain architecture suggests a conserved structural foundation essential for basic HKT function, likely established early during vascular plant evolution. This conservation may indicate strong selective pressure to maintain a specific molecular mechanism for sodium or potassium transport critical for physiological processes like osmoregulation or xylem loading in a terrestrial environment. In monocotyledons, not only the number of HKT sequences was large, but also the HKT proteins of TrkH x2 type of domain architecture were more than the TrkH x1 type. The TrkH x2 type proteins, which contain two pore-forming domains, likely provided monocots with enhanced regulatory flexibility and functional specificity (Fig. 5C). Subcellular localization analysis revealed that the majority (134 proteins) were predominantly localized to the plasma membrane. Based on PDBe database, all HKT proteins were predicted to have seven structures, including three crystal structure and four crystal structure of transport complex (Figs 5D and S7). Amino acid sequence variations in the HKT transporters of TrkH domain influence their tertiary protein structure (Fig. S7). The *A. thaliana* mutant for the *hkt* gene exhibits a markedly attenuated capacity to withstand salt stress. This deficiency could be primarily attributed to the disruption of ion homeostasis, leading to exacerbated sodium toxicity and compromised osmotic adjustment (Fig. S8). The molecular function of these proteins had cation transmembrane transporter activity (GO:0008324) and mediated cation transport (GO:0006812) and transmembrane transport (GO:0055085) (Fig. 5E).

**Figure 6:**
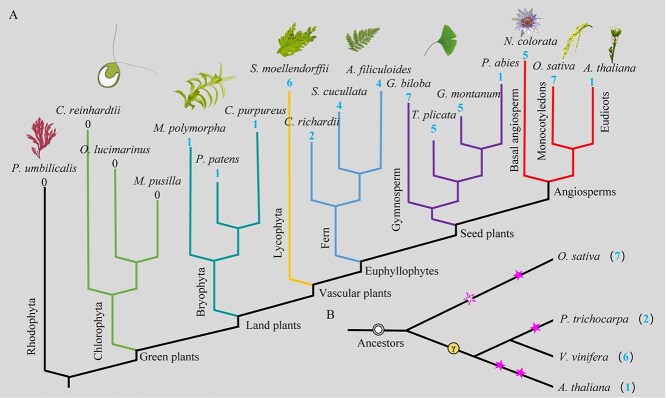
The phylogenetic relationships of species and HKT proteins distribution. A. The phylogenetic relationship of plant species from rhodophyta to angiosperms and the distribution of HKT proteins in some plants in this study. B. The phylogenetic relationships of representative angiosperm species and the distribution of HKT proteins. The pentagram represented the WGD and the circle represented the whole-genome triplication.

Plant evolution is fundamentally shaped by environmental pressures, driving the development of adaptive traits that enhance survival and reproductive success. Over geological timescales, plants have undergone significant morphological, physiological, and genetic modifications in response to abiotic (e.g. climate, soil, water availability) and biotic (e.g. herbivory, competition) factors. As the first landed higher plants, bryophytes began to evolve HKT proteins with the TrkH domain, with only one member in each species (Fig. 6A). Subsequently, the HKT gene family was expanded in the evolutionary process of plants. Different from the seed plant evolution, each lineage from the nonseed plants evolved independently according to the characteristics of HKT sequences (Fig. 1). Meanwhile, HKT proteins had similar evolutionary features in gymnosperm and basal angiosperm. One exception was that the SmoHKT6 protein of *S. moellendorffii* was classified into a branch of gymnosperms (Fig. 2A). The evolution of *SmoHKT* genes to represent lycophytes might have limitations because only *S. moellendorffii* genome had been completed. On the whole, we found convergent evolutionary patterns between HKT protein diversification and species divergence trajectories. We therefore inferred that *HKT* genes contributed to species divergence. The copies of the *HKT* gene varied greatly in angiosperms, especially eudicots (Figs 5B and 6). Here, we found that *HKT* gene copies were closely related to polyploidy events in species (Fig. 6B). The contraction event of *HKT* genes occurred every time the plant undergoes a WGD event after the ancient triplication.

## Discussion

### Distribution of the HKT gene family in different phylogenetic lineages

The evolution of plants follows the direction from simple to complex, from aquatic to terrestrial. Meanwhile, green plants first originated in marine deep-water environments and later colonized fresh water and dry land [55]. HKT transporter is the main carrier protein for transporting the cation in the plant cells, which is closely correlated with cell osmotic pressure. In previous studies, the identification of HKT sequence is only carried out in *P. patens*, which belong to bryophytes [5, 13]. In this study, we have identified HKT sequences in different phylogenetic lineages from algae to angiosperms. Interestingly, HKT sequences are not detected in the ancient plants rhodophyta and chlorophyta. As the first landed higher plants, bryophytes not only have *HKT* genes but also only one. Even with different retrieval strategies, *HKT* genes are found in each vascular plant, including lycophytes and euphyllophytes [1, 13, 56, 57]. This means that the *HKT* gene is generated with the landing of the plant.

Given the rapid accumulation of genomic data, we have known that there exist gene number differences among organisms, just in the same genome, the different genes or gene families are various in number. In vascular plants, more than 50% of genes have more than 10 copies, while in lower plants, the high copy genes are significantly less than in vascular plants, and most genes have only 1 or 2 copies [58]. Here, we found that there are differences in the number of *HKT* genes among different phylogenetic lineages. In gymnosperms, *P. lambertiana* and *G. biloba* genomes have 12 and 11 chromosomes, containing 38 518 and 27 832 protein-coding genes, with a size of 28.96 and 9.87 Gb, respectively [59, 60]. In Rosaceae, *M. domestica*, and *P. persica* genomes have 17 and 8 chromosomes, containing 42 140 and 27 852 protein-coding genes, with a size of 647.51 and 214.22 Mb, respectively [61, 62]. The genome size of *C. reinhardtii* is about 120 Mb, and the number of genes is about 18 000, which is far more than that of other single-cell green algae [63]. The results show that a significant correlation does not exist between the number of *HKT* genes in plants and physical information of the genome, including genome size, chromosome number, and protein-coding genes. Phylogenetically informed comparative methods confirmed the absence of significant associations between HKT gene copy number and core genomic parameters, implying that lineage-specific selection pressures, rather than WGD history or karyotype evolution, predominantly shape HKT family diversity. Previous studies raised that the independent multiplication of *HKT* genes in *O. sativa* and *S. moellendorffii* may be correlated with the affinity of these vascular plants to moisture environments [13]. In addition to *O. sativa* and *S. moellendorffii,* the *HKT* genes of *N. colorata* and *N. thermarum* also have this feature in our study. However, exceptions to this feature was found, such as *G. biloba*, *G. montanum,* and *B. distachyon*. Studies on tetraploid *P. virgatum* showed a positive correlation between chromosome ploidy and the number of *HKT* genes. Our analysis indicates that the closely related species have similar *HKT* genes distribution, especially within the genus. Adaptive evolution typically involves gene family expansion, epigenetic modifications, and horizontal gene transfer, with salt-tolerant HKT transporters serving as a representative example.

The WGD and whole-genome triplication events are remarkable characteristics of the plant genome [64]. Following an ancient triplication event, the *V. vinifera* genome has not experienced additional recent WGDs [65], whereas the genomes of *P. trichocarpa* and *A. thaliana* have undergone one and two such events, respectively [66–68]. Here, six, two and one *HKT* genes are found in *V. vinifera*, *P. trichocarpa,* and *A. thaliana*, respectively. *P. communis* and *M. domestica* experience two WGD events, that is, the ancient triplication event and the recent WGD event, which occurred before the differentiation of *P. communis* and *M. domestica* [61, 69]. Only one *HKT* gene is found in *P. communis* and *M. domestica*, respectively. In eudicots, some *HKT* genes are lost during the evolution of reconstructed diploids. Previous studies have also found that a series of genetic events during the diploidization of the genome after duplication lead to genes and chromosome reduction [70, 71]. So far, whole-genome triplication event has occurred only in eudicots, but not in monocotyledons. *O. sativa* studies show that the WGD event is occurred in the genome and appears before the differentiation of Poaceae plants [72–75]. In addition to the shared Poaceae WGD event, *Z. mays* experiences an intra-genus WGD event [76, 77]. *Z. mays* originates from allotetraploid, which is considered to have a heterologous duplication process after its differentiation with *S. bicolor* [78]. In this study, seven, four, and three *HKT* genes are found in *O. sativa*, *S. bicolor,* and *S. bicolor*, respectively. The duplication event of the genome is a potential factor in the change of the *HKT* gene copies. Although WGD plays a key role in the adaptive evolution of angiosperms [79, 80], few recent WGD events are found in existing gymnosperms [81, 82]. The genetic relics support one ancient WGD event in gymnosperms, which is considered to be a polyploidization event in the ancestors of all extant seed plants [60, 82–84]. Pinaceae and Cupressaceae independently have an ancient WGD event [81, 82], but there is no additional round of lineage-specific WGD event in the genome of *G. biloba* [60]. Our genomic analysis reveals that *G. biloba* exhibits the highest copy number of *HKT* genes among the species examined, whereas members of the Pinaceae and Cupressaceae families possess comparatively fewer copies, a trend particularly pronounced within the Pinaceae lineage. The genetic relics support two ancient WGD events in ferns, of which a recent WGD event occurred in *A. filiculoides* following its divergence from *S. cucullata* [60]. The same number of *HKT* genes were identified in *A. filiculoides* and *S. cucullata*, but only two *HKT* genes were identified in *C. richardii*, belonging to homosporous fern. Because the *C. richardii* genome is only partially assembled [85], the copies of *CriHKT* are not representative. The genome of *M. polymorpha* has not undergone the common ancient WGD event, while the genome of *P. patens* has undergone two WGD events [86–88]. We find one *HKT* gene in *M. polymorpha* and *P. patens,* respectively, so the WGD event does not affect the copy of the *HKT* gene in bryophytes.

The tandem duplication represents the predominant mechanism for HKT gene family expansion in species harboring multiple copies (*n* > 2). According to the descriptions of Holub, a chromosomal region within 200 Kb containing two or more paralogous genes is defined as a tandem duplication event [89], and there will be more tandemly duplicated *HKT* genes in plants. The HKT gene family forms gene clusters in part of eudicots, such as *V. vinifera*, *F. vesca,* and *P. mume*. The same results are obtained in previous studies on *HKT* genes of five Rosaceae plants [90]. A gene cluster is two adjacent genes produced by duplication or hundreds of genes arranged in tandem. Species may have a large number of tandemly duplicated genes that require a large number of gene products, such as rRNA genes and histone genes [91, 92]. Tandem duplication of gene clusters is the result of gene duplication caused by DNA fragment mismatch during genome replication and the repair mechanism is not activated [93, 94]. Duplication events also occur in functional domains within genes [95, 96]. In this study, 134 HKT proteins from fifty species have two types of domain architectures (TrkH x1 or TrkH x2). Tandem functional domains have profoundly influenced the evolution of genes. HKT sequences with similar structures are clustered into a branch in different phylogenetic lineages.

## Materials and methods

### Gene discovery

In order to obtain as many as *HKT* genes in sequenced plant genomes, we identified the coding and genomic sequences of *HKT* genes in fifty species with complete genomes using several datasets. These species included one rhodophyte, three chlorophytes, three bryophytes, one lycophyte, and forty-five euphyllophytes. The detailed information of the species was shown in Table S1. The Hidden Markov Model (HMM) profile of the TrkH domain (PF02386) was downloaded from the Pfam database (v35.0) [3, 15] and used in local searches of the datasets. Hmmsearch from the HMMER project (v3.3.2) was employed to identify HKT sequences of plants, with a threshold of *e*-value < *e*^−5^ [34]. Then, we screened all putative HKT sequences to confirm the presence of the TrkH domain using SMART database again [35]. Meanwhile, we removed subsequently the sequences with ≥95% amino acid identity using the cd-hit software [36]. Finally, the confirmed sequences were used for further analysis.

### Comparative genomic analyses

To understand the evolutionary position of early species, we selected ten species for comparative genomic analysis, including rhodophyta, chlorophyta, bryophytes, lycophytes, ferns, and gymnosperms. We used the MCL inflation of default parameters (v1.5) as the cluster granularity setting [37]. The Mafft (v7) was employed for sequence alignment, with a strategy FFT-NS-2 [38]. Orthologous groups were identified from ten early species using OrthoFinder [39]. We constructed gene trees of all the orthologous groups and a species tree using FastTree [40]. Divergence times among the plant species were estimated based on molecular dating data obtained from the TimeTree database [41].

### Phylogenetic reconstruction

Phylogenetic analyses were conducted using two methods: neighbor joining (NJ) and maximum likelihood (ML) [42, 43]. The HKT sequences were aligned using MUSCLE (v3.8.31) with default parameters [44]. Then, NJ trees were constructed based on the multiple sequence alignment files using Mega (v7.0) with Poisson correction model and 1000 bootstrap resampling, pairwise deletion option [45]. Meanwhile, the Mafft (v7) was employed for sequence alignment, with a strategy G-INS-1 [38]. Phylo (v1.0) was employed to construct ML trees, with the Jones, Taylor, and Thorton (JTT) model, ignoring heterogeneity among sites and 100 resampling [46]. Meanwhile, ML trees were constructed using RAxML (v8.2.4) with 100 nonparametric bootstrap replicates, the JTT model, and gamma distribution option [47]. The phylogenetic tree for this family’s seed alignment was constructed using FastTree [40]. We used FastTree to calculate neighbor join trees with a local bootstrap based on 100 resamples (shown next to the tree nodes). FastTree calculated approximately-maximum-likelihood phylogenetic trees from our seed alignment.

### Chromosome localization and synteny analyses

The annotation files of the genomes were downloaded from the database corresponding to the genome of the species. We obtained the information on the chromosomes and gene location. Then, the physical locations of *HKT* genes were visualized using TBtools software [48]. Whole-genome synteny analysis was conducted by pairwise protein sequence comparison, employing BLASTp algorithm (v2.8.1) with stringent parameters (−*e-*value 1*e*-10 − num_threads 64 − outfmt 6 − num_alignments 5) for multiple sequence alignment, ensuring detection of evolutionarily conserved gene blocks [49]. The tandem and collinearity genes were identified using MCScanX software with alignment significance defined according to an *E*-value <1*e* − 05 [50].

### Structure exploration and gene ontology annotations

Search tool from the Pfam database (v35.0) was employed to identify architectures of all TrkH domain proteins, with a threshold of *E*-value = 1.0 [15]. The FASTA-format file containing HKT sequences was uploaded to search for matching Pfam families using the HMMER website. Protein Data Bank in Europe (PDBe) was used to predict the structure characteristics of different HKT proteins [51]. DeepTMHMM and Phobius were used to analyze transmembrane helices [52, 53]. Comprehensive functional profiling of HKT protein sequences was performed through computational interrogation of the Gene Ontology resource at EMBL-EBI, employing semantic similarity metrics to identify statistically enriched (FDR <0.05) GO terms spanning cellular localization, molecular activity, and biological pathway annotations [54].

### Expression pattern and functional analysis of genes

*V. vinifera* occupied a unique evolutionary position among plants. In our study, we identified that grapevine contained a relatively large number of *HKT* genes exhibiting distinct structural features, which prompted its selection as our experimental material. We collected transcriptome datasets from 54 grape samples, covering most organs across different developmental stages for tissue-specific expression pattern analysis. The sampled plant organs included: buds, inflorescences, tendrils, leaves, stems, roots, developing berries, withered berries, seeds, rachises, anthers, carpels, petals, pollen, and seedlings (NCBI BioProject: PRJNA152939). Select one-year-old asexually propagated seedlings with uniform growth vigor for salt stress and drought stress treatments. Salt stress treatment was performed by applying 100 mM NaCl for 0, 3, 6, 12, and 24 h, respectively. For drought stress, the initial condition was established by watering the pots to maintain soil relative water content above 50%, after which watering was withheld to induce drought stress. Samples were collected at 0 d, 8 d, 16 d, and 24 d of drought treatment. Primers for gene cloning were designed using the Primer Premier 5.0 software (Table S2). The relative gene expression levels were quantified using the CFX96 Real-Time PCR Detection System (Bio-Rad, Hercules, CA, USA), with *VviActin* serving as the reference gene.

The *A. thaliana* wild-type and *hkt* mutant materials used in this study were all in the Columbia-0 genetic background. Following surface sterilization in 10% NaClO for 20 min, the seeds were rinsed thoroughly with distilled water a minimum of three times. Subsequently, they were subjected to a 3 d at 4°C. Germination was carried out on ½ Murashige and Skoog (½ MS) medium containing 1.5% (w/v) sucrose, under controlled conditions: 22°C, 60%–80% relative humidity, and a 16 h/8 h light/dark cycle. For the salt stress assay, seeds were germinated on ½ MS medium for 3 d and then transferred to ½ MS medium supplemented with 0.1 M NaCl for 15 d before phenotypic observation.

## Supplementary Material

Web_Material_uhaf245

## Data Availability

Detailed information and genome download sites for fifty species were collected in Table S1.
