# Supplementary material for: The origin and evolution of HKT proteins with TrkH domain from aquatic plants to flowering plants
Source: Hortic Res. 2025 Sep 15;12(12):uhaf245. doi: 10.1093/hr/uhaf245 (PMC12703196; doi:10.1093/hr/uhaf245)
Supplement: Web_Material_uhaf245 [file web_material_uhaf245.zip › Supplementary Figures.docx]

**Supporting information**

**
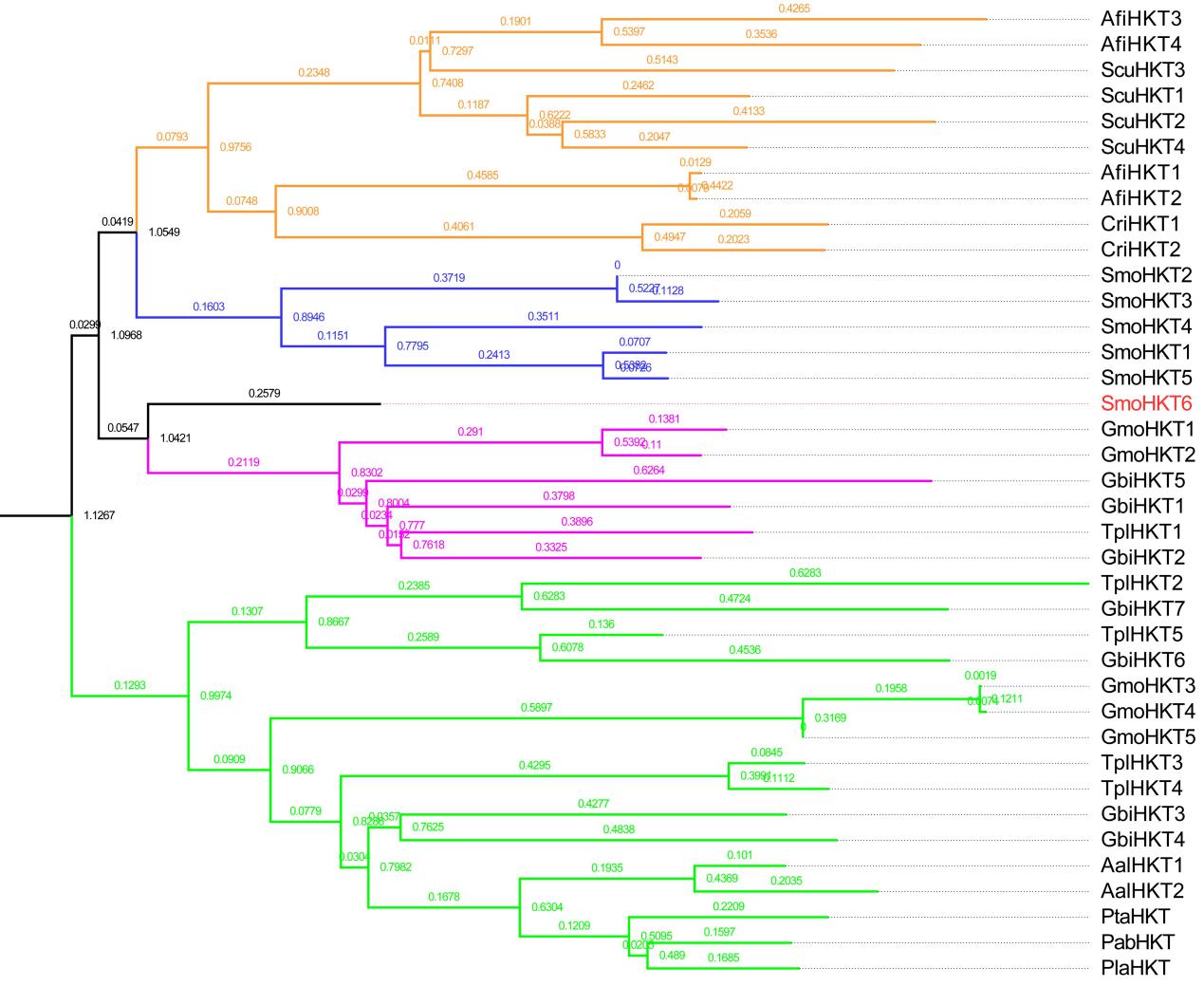
**

Figure S1. Phylogeny of HKT gene family in six gymnosperms, three ferns and one lycophytes. The corresponding species and gene IDs for each gene are provided in Supplementary Table S3.


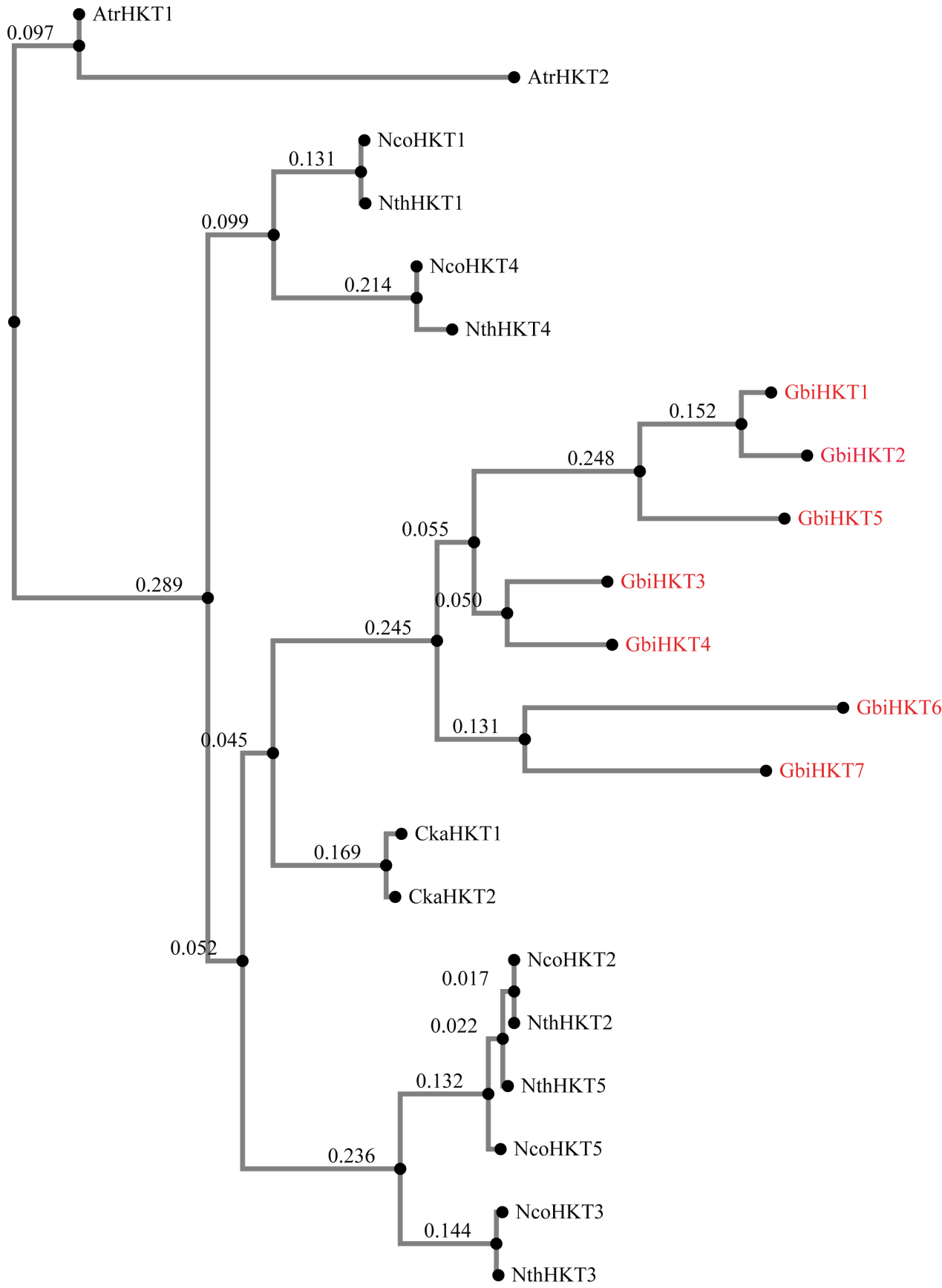


Figure S2. Phylogeny of HKT gene family in *G. biloba* and basal angiosperms. The corresponding species and gene IDs for each gene are provided in Supplementary Table S3.


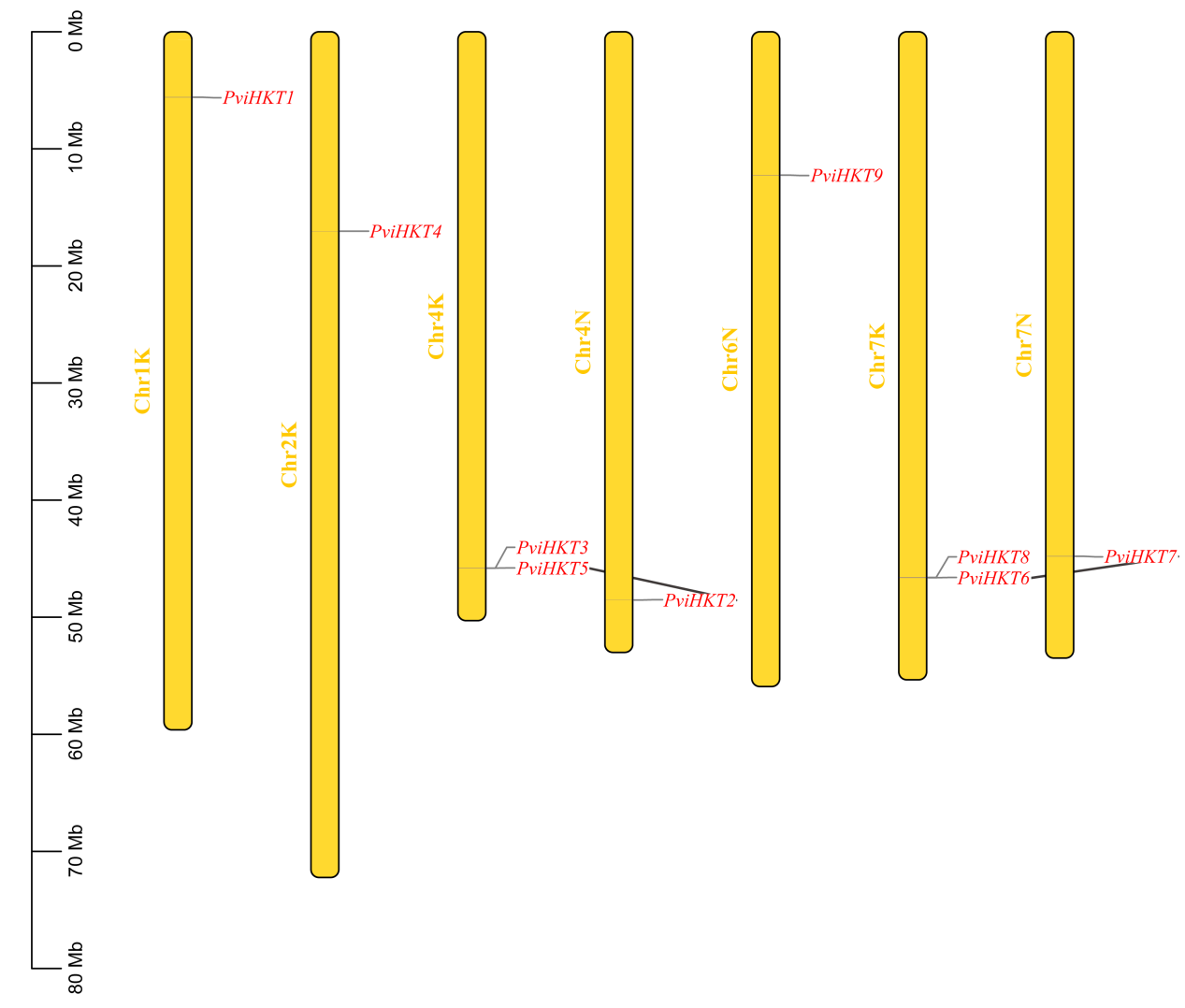


Figure S3. Chromosomal localization and duplication events of HKT gene family in *P. virgatum*.


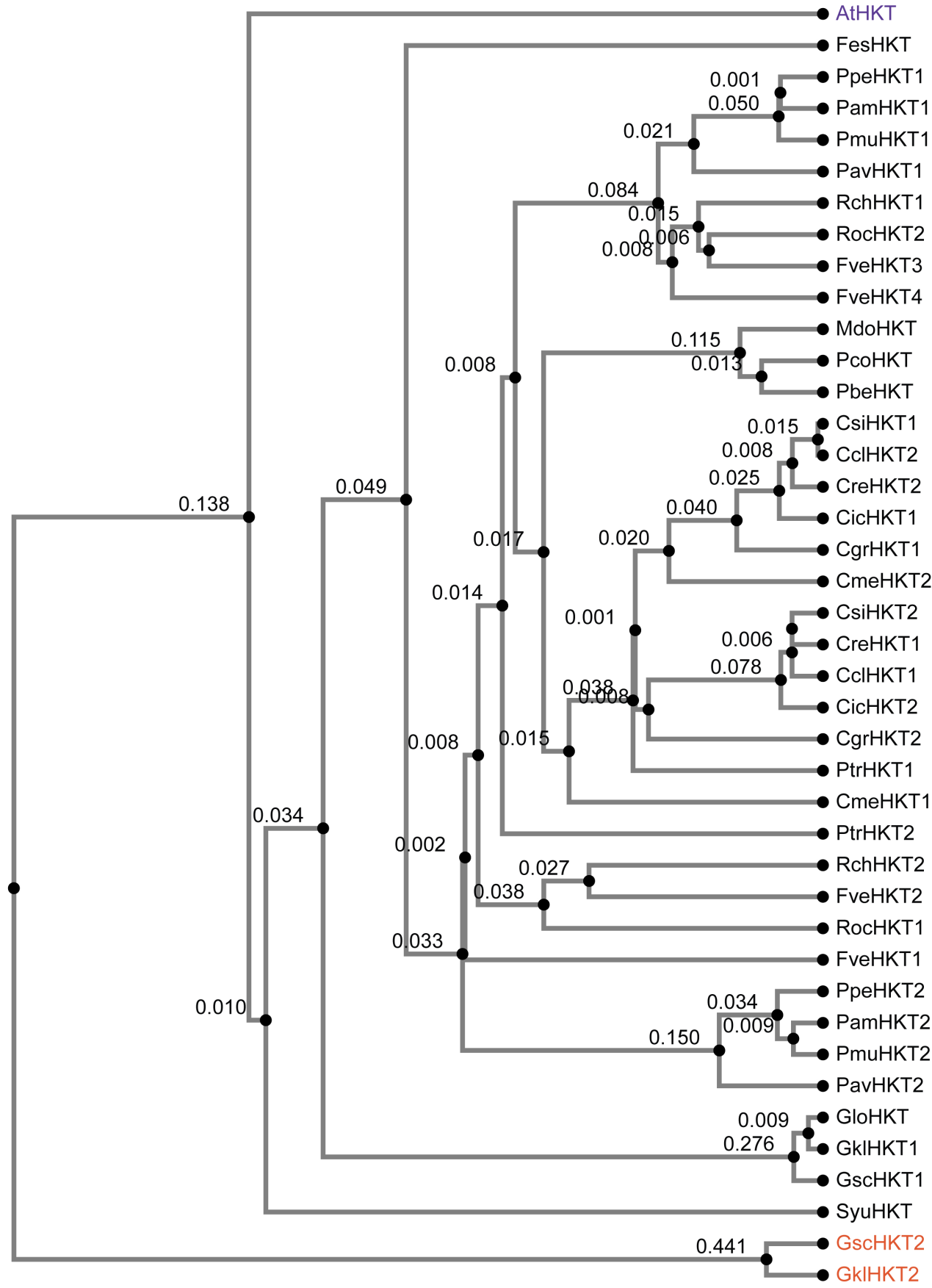


Figure S4. Phylogeny and number of HKT gene family in fourteen dicotyledons. The corresponding species and gene IDs for each gene are provided in Supplementary Table S3.


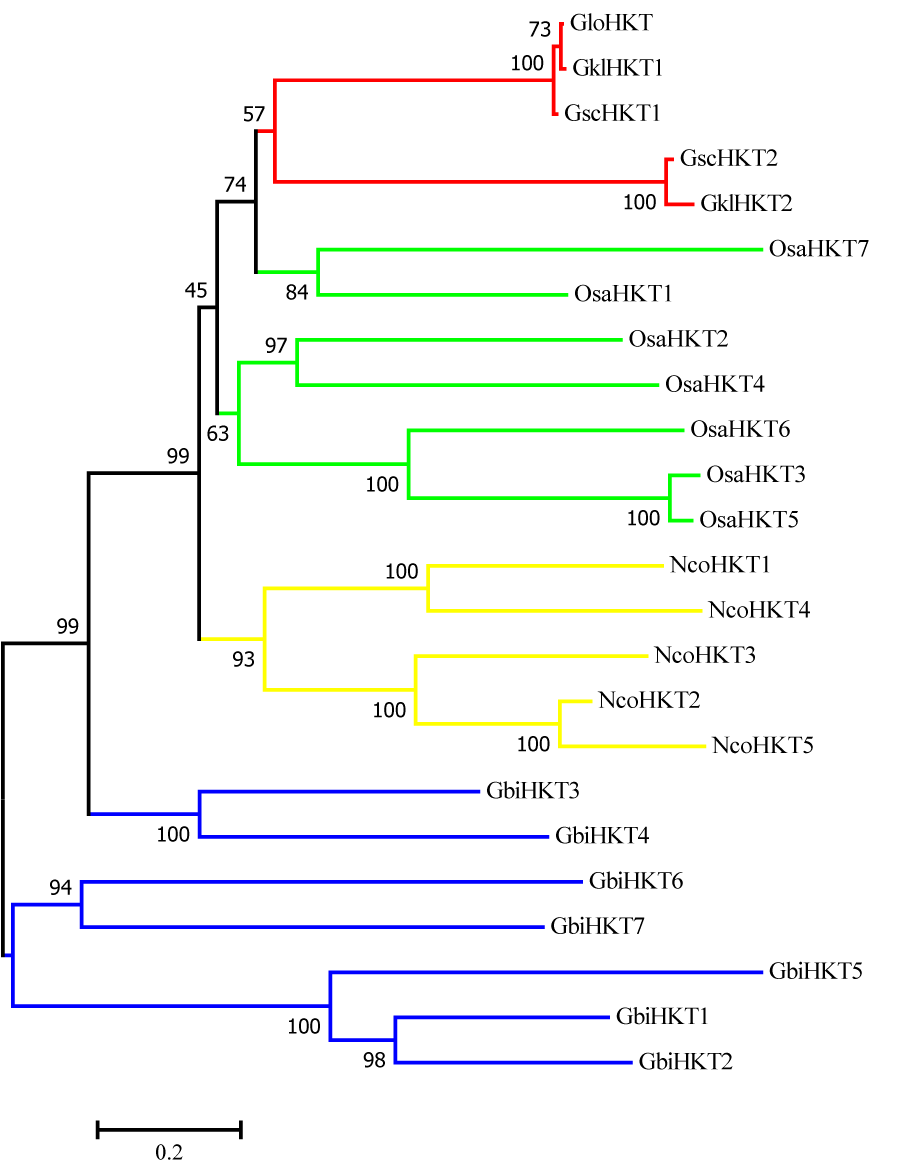


Figure S5. Phylogeny of HKT gene family in *G. lobatum, G. schwendimanii, G. klotzschianum, O. sativa, N. colorata* and *G. biloba*. The corresponding species and gene IDs for each gene are provided in Supplementary Table S3.


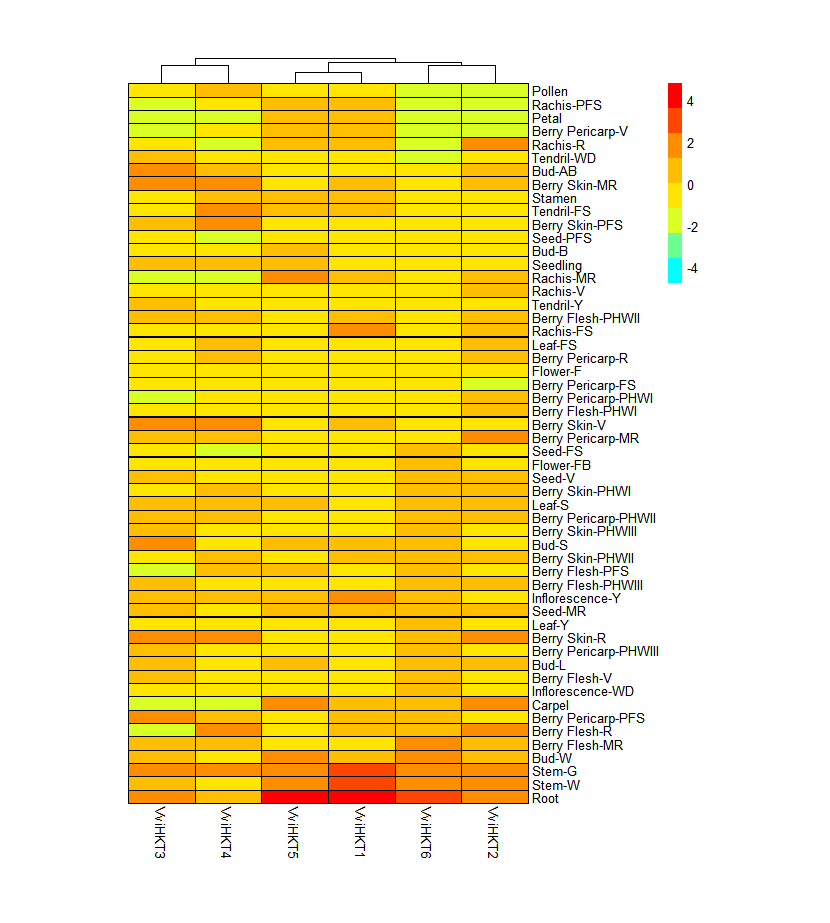


Figure S6. The expression patterns of *VviHKT* genes across 54 grapevine tissues and organs.


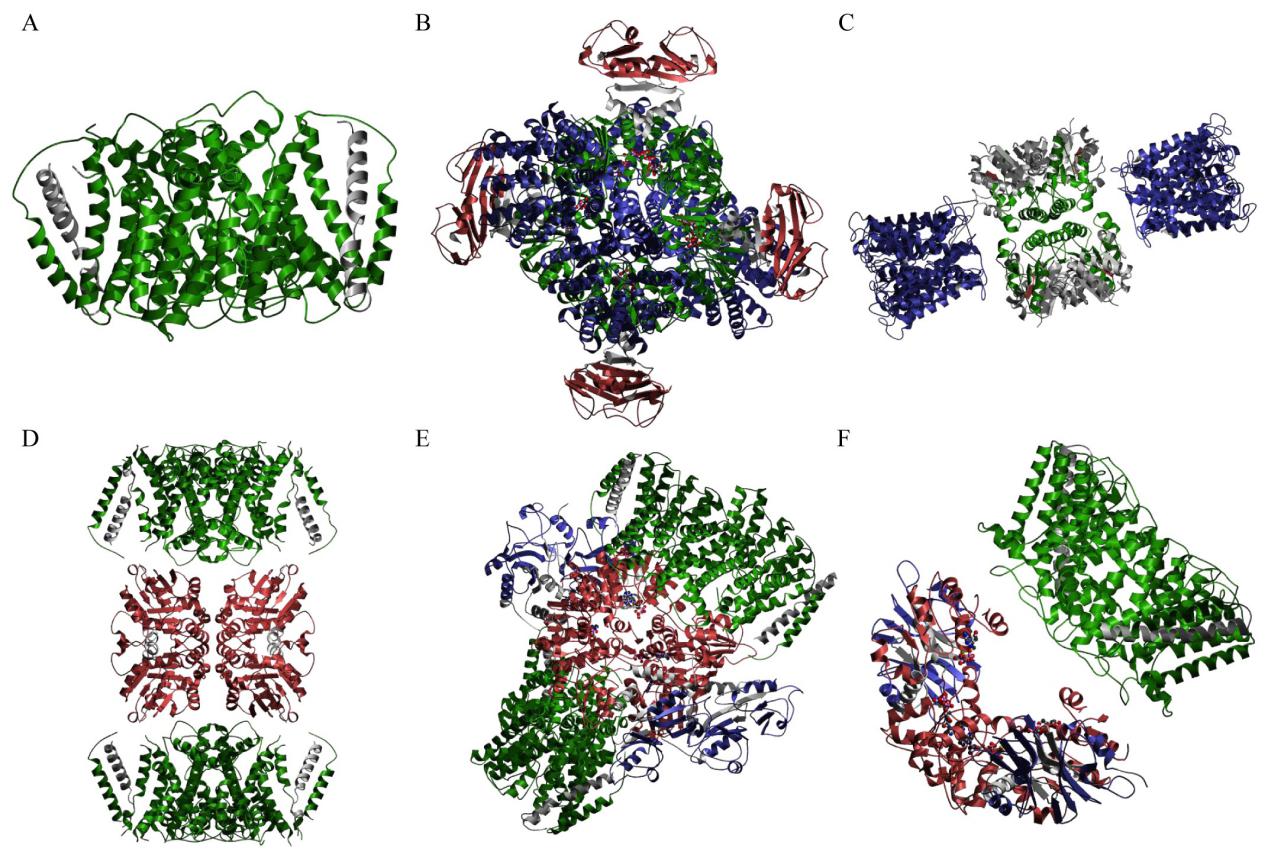


Figure S7. Prediction of HKT protein structure from fifty species. A. Crystal Structure of the potassium transporter TrkH from *Vibrio parahaemolyticus* (PDB entry 3PJZ). B. KtrAB potassium transporter from *Bacillus subtilis* (PDB entry 4J7C). C. Crystal structure of inactive conformation of KtrAB K^+^ transporter (PDB entry 5BUT). D. Structure of TrkH-TrkA in complex with ATP (PDB entry 6V4J). E. Structure of TrkH-TrkA in complex with ADP (PDB entry 6V4K). F. Structure of TrkH-TrkA in complex with ATPgammaS (PDB entry 6V4L).


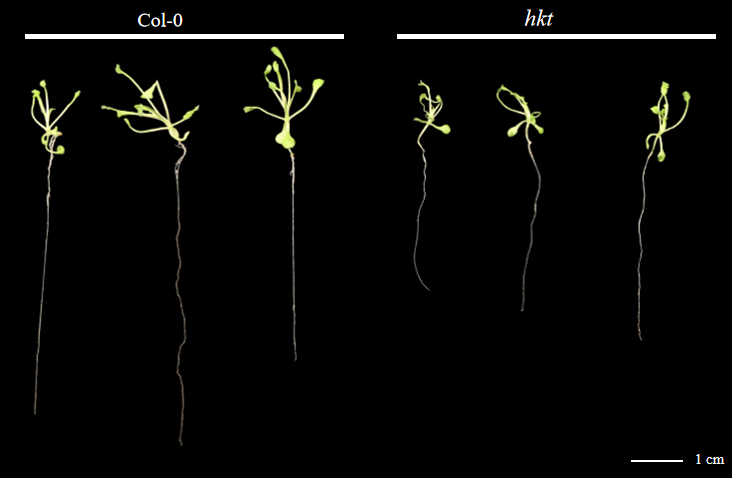


Figure S8. The phenotypic characteristics of *A. thaliana hkt* gene mutant plants in response to salt stress. Col-0 refers to *A thaliana* plants of the Columbia-0 background, while *hkt* denotes *A. thaliana* mutant plants for the *hkt* gene.
